# Diffusion Tractography Biomarker for Epilepsy Severity in Children With Drug‐Resistant Epilepsy

**DOI:** 10.1002/acn3.70217

**Published:** 2025-10-08

**Authors:** Jeong‐Won Jeong, Min‐Hee Lee, Hiroshi Uda, Yoon Ho Hwang, Michael Behen, Aimee Luat, Csaba Juhász, Eishi Asano

**Affiliations:** ^1^ Department of Pediatrics Wayne State University Detroit Michigan USA; ^2^ Translational Imaging Laboratory University Health Center Detroit Michigan USA; ^3^ Department of Neurology Wayne State University Detroit Michigan USA; ^4^ Translational Neuroscience Program Wayne State University Detroit Michigan USA; ^5^ Department of Pediatrics Central Michigan University Mt. Pleasant Michigan USA

## Abstract

**Objective:**

To develop a novel deep‐learning model of clinical DWI tractography that can accurately predict the general assessment of epilepsy severity (GASE) in pediatric drug‐resistant epilepsy (DRE) and test if it can screen diverse neurocognitive impairments identified through neuropsychological assessments.

**Methods:**

DRE children and age‐sex‐matched healthy controls were enrolled to construct an epilepsy severity network (ESN), whose edges were significantly correlated with GASE scores of DRE children. An ESN‐based biomarker called the predicted GASE score was obtained using dilated deep convolutional neural network with a relational network (dilated DCNN+RN) and used to quantify the risk of neurocognitive impairments using global/verbal/non‐verbal neuropsychological assessments of 36/37/32 children performed on average 3.2 ± 2.7 months prior to the MRI scan. To warrant the generalizability, the proposed biomarker was trained and evaluated using separate development and independent test sets, with the random score learning experiment included to assess potential overfitting.

**Results:**

The dilated DCNN+RN outperformed other state‐of‐the art methods to create the predicted GASE scores with significant correlation (*r* = 0.92 and 0.83 for development and test sets with clinical GASE scores) and minimal overfitting (*r* = −0.25 and 0.00 for development and test sets with random GASE scores). Both univariate and multivariate models demonstrated that compared with the clinical GASE scores, the predicted GASE scores provide better model fit and discriminatory ability, suggesting more adjusted and accurate estimate of epilepsy severity contributing to the overall risk.

**Interpretation:**

The proposed biomarker shows strong potential for early identification of DRE children at risk of neurocognitive impairments, enabling timely, personalized interventions to prevent long‐term effects.

## 1 Introduction

Neurocognitive impairments are prevalent in children with drug‐resistant epilepsy (DRE). This highlights the importance of neurocognitive screening, diagnosis, and management to optimize their academic achievement and well‐beings [1, 2, 3]. Complex and multifaceted risk factors are involved in the occurrence of these impairments, including but not limited to, lifetime epilepsy duration, specific epilepsy syndrome, location of epileptogenic focus, seizure type/severity, number of antiseizure drugs (ASD), and interictal abnormalities [4, 5, 6]. Therefore, their influence on epilepsy severity becomes complex and challenging for clearly understanding the impact of epilepsy severity on the clinical management of neurocognitive impairments.

Global Assessment of Severity of Epilepsy (GASE) has been considered a quick and simple metric that could be used as an overall measure of epilepsy severity in pediatric epilepsy [7, 8]. GASE is a single overall epilepsy severity score that can be obtained from parent or caregiver inputs, along with other more structured questionaries [9, 10, 11, 12]. Although it could help quantify epilepsy severity as a broad assessment, it is inherently limited by subjective judgment that introduces variability across clinicians or caregivers, leading to inter‐rater inconsistency and issues with standardization. More importantly, the lack of consideration of the findings of neuroimaging data could reduce its precision and fail to leverage available clinical information for a more comprehensive severity estimate.

Numerous neuroimaging studies [13, 14, 15, 16] have examined the effect of epilepsy on the development of gray matter and white matter, and its association with neurocognitive impairments. Notably, our recent study [17] using diffusion‐weighted MRI (DWI) tractography presented supporting evidence indicating that seizure onset zones in DRE children have poorly pruned axonal connections indicating delayed maturation of white matter tracts that could contribute to more widespread and frequent seizures. Building on these findings, our subsequent DWI tractography studies [18, 19] demonstrated that such seizure‐associated connectivity abnormalities are significantly associated with the reduced efficiency of neighboring connections in diverse language networks that could be used as a quantitative imaging marker for predicting the severity of language impairment in DRE children.

Based on our prior findings, this study introduces a novel deep learning (DL)‐based preoperative evaluation framework that leverages clinical DWI tractography data to construct an epilepsy severity network (ESN) using the clinician‐assigned GASE scores, allowing the extraction of a new imaging‐based epilepsy severity biomarker—specifically, the predicted GASE score by using a dilated deep convolutional neural network with a relational network (dilated DCNN+RN) [20, 21]. The predicted GASE score is then used to estimate the potential risk of neurocognitive impairments, quantified as a hazard ratio (HR) using the Cox proportional hazards model [22, 23]. The central hypothesis is that preoperative DWI tractography data would provide an effective imaging biomarker for predicting the severity of epilepsy‐related cognitive burden, reflected by the clinician‐assigned GASE score (i.e., gold standard outcome assessed for epilepsy severity).

## 2 Method

### 2.1 Subjects

The present study included 51 children clinically diagnosed with DRE (age: 11.8 ± 3.4 years, 24 boys, Table S1) who underwent two‐stage resective surgeries at the Children's Hospital of Michigan, Detroit, between 2009 and 2021. These patients were selected by applying the following inclusion criteria: (1) age: 2.5–19 years; (2) a history of medically refractory focal epilepsy scheduled for intracranial EEG (iEEG) recording as a part of the pre‐surgical evaluation; and (3) preoperative GASE assessment and MRI acquisition including DWI tractography scan were successfully completed. Exclusion criteria consisted of the following: (1) presence of massive brain malformations (such as large perisylvian polymicrogyria or hemimegalencephaly), which could cause early functional and structural reorganization of language functions; (2) history of previous neurologic surgery; and (3) autism spectrum disorder based on caregiver report and direct observations. In addition, 29 healthy children (age: 11.6 ± 3.3 years, 14 boys) were enrolled as a control group, matched to the DRE children by age, sex, and MRI protocol.

It should be noted that our study cohort (*n* = 80) was divided into two separate sets, (1) development set (*n* = 55, 35 DRE; 20 control) to train and validate the performance of the proposed DL model and (2) independent test set (*n* = 25, 16 DRE; 9 control) to validate the generalizability of the trained DL model on an independent study cohort that was not included in the model development. The two sets were statistically matched for age and sex using standardized mean differences (SMD) [24] to ensure comparability (i.e., SMD values of age and sex were 0.014 and 0.015, respectively). The Institutional Review Board approved this study, and written informed consent or a waiver of consent was obtained from the patients and/or their guardians of the patients.

### 2.2 Evaluation of GASE Score

Two clinicians (A.L., H.U.) jointly agreed on the GASE scale score assigned to each participant in our DRE cohort. The resulting score was then used as a ground‐truth measure of epilepsy severity. Briefly, the GASE scale rates the overall severity of epilepsy on a 7‐point Likert scale, where 1 indicates “not at all severe” and 7 indicates “extremely severe”. The rating is based on a comprehensive clinical impression that incorporates: (1) seizure frequency, (2) intensity of seizures, (3) fall or injuries during seizures, (4) severity of the post‐ictal period, (5) amount of ASD, (6) side effects of ASD, and (7) interference of epilepsy or drugs in daily activities [8]. The GASE score for each of the participants was rated based on seizure frequency (daily, weekly, and monthly), the presence of associated drop or falling episodes leading to injuries during the seizure as well as the length and severity of the post‐ictal state. The ASD of each participant, including the total number, the amount in mg/kg/day, and the presence of associated side effects based on parents or caregivers' reports, were noted. In both development and test datasets, we assumed the clinician‐assigned GASE score of 7 for each control participant, which is the minimum score in the range of 7–49.

### 2.3 Evaluation of Neurocognitive Impairment

Global, verbal, and nonverbal intellectual functioning was assessed using the age‐appropriate versions of the Weschler series; the Wechsler Preschool and Primary Scale of Intelligence (WPPSI) for children 2.6 through 6.0 years of age, the Wechsler Intelligence Scale for Children (WISC) for children 6 years through 16 years of age, and the Wechsler Adult Intelligence Scale (WAIS) for individuals older than 16 years of age. According to the Wechsler classification scheme, individuals' performance in class I: general cognitive ability, class II: verbal ability, and class III: non‐verbal ability was then categorized into: score (1) high average (Standard score [SS] > 110); score (2) average (SS = 90–110); score (3) low average (SS = 80–89); score (4) borderline (SS = 70–79); score (5) mildly impaired (SS = 60–69); score 6) severely impaired (SS < 60). It is important to note that missing data in the study cohort limited the analysis to only 36, 37, and 32 DRE patients who completed age‐appropriate assessments to identify the neurocognitive impairment in global, verbal, and nonverbal intellectual functioning, respectively. Their time interval between MRI and neuropsychological assessment was 3.2 ± 2.7 months.

### 2.4 MRI Acquisition

Clinical brain MRI data of all participants were obtained using a 3 T GE Signa scanner (GE Healthcare, USA). DWI tractography data was acquired with the following parameters: repetition time (TR) = 12,500 ms, echo time (TE) = 86.4 ms, flip angle = 90°, slice thickness = 3 mm, 55 isotropic gradient directions at *b* = 1000 s/mm^2^, and single *b* = 0 image acquisition. T1‐weighted images were also obtained with the following parameters: TR = 6.1 ms, TE = 2.1 ms, flip angle = 12°, and slice thickness = 1.2 mm. Diverse DWI artifacts including head motion, noise, physiological artifacts, susceptibility‐induced distortion, B1 field inhomogeneity, and eddy current‐induced distortion were corrected using the FSL tools [25, 26] and a DL‐based distortion correction approach [27].

### 2.5 Imaging‐Based Biomarkers for Epilepsy Severity

**FIGURE 1:**
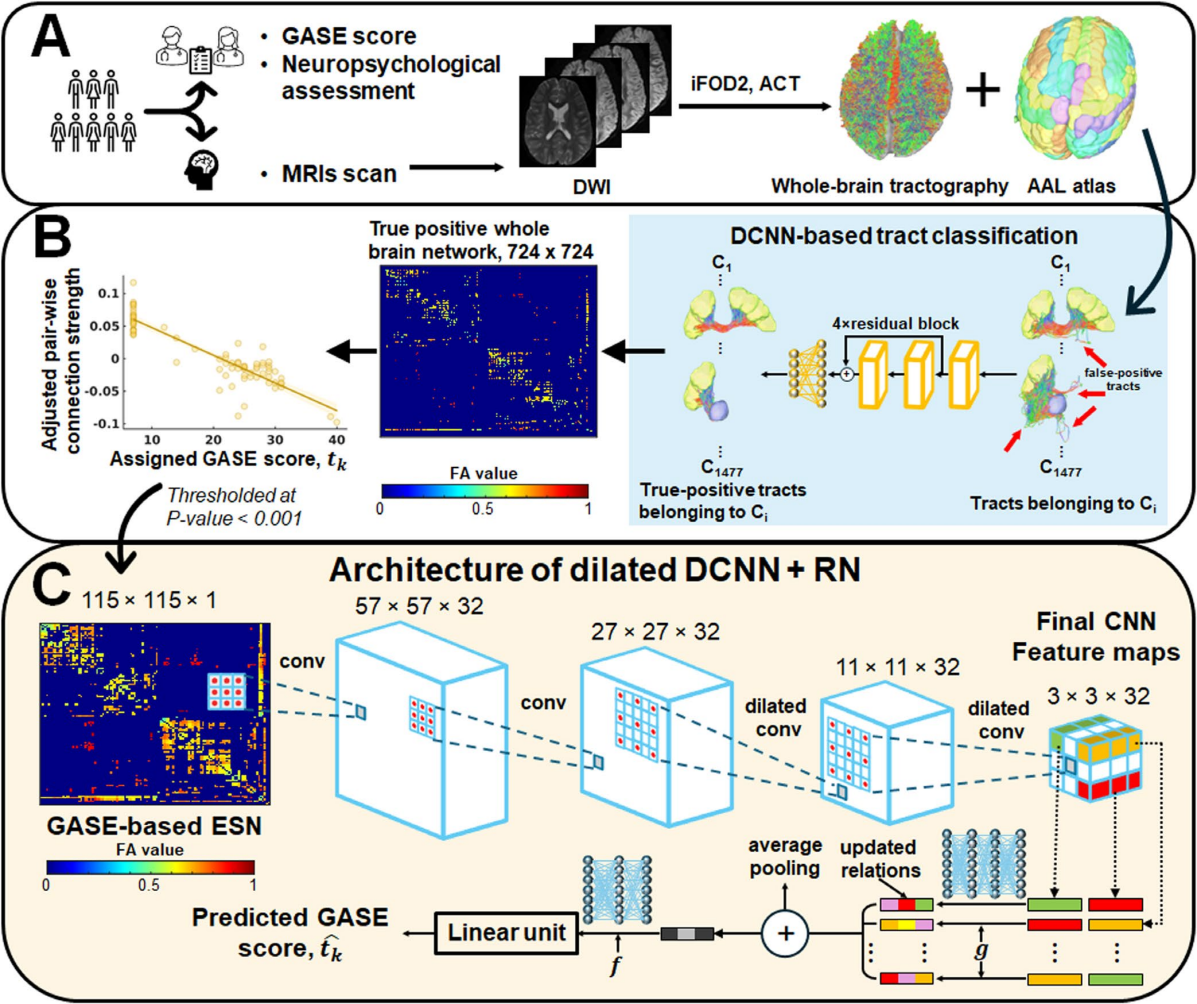
Detailed architecture of the proposed DL‐based preoperative evaluation framework that utilizes clinical DWI tractography scan of DRE children. (A) Construction of whole‐brain tractography. (B) Construction of epilepsy severity network (ESN) from true positive whole‐brain network and clinician‐GASE score. (C) Imaging‐based prediction of GASE score using ESN and dilated deep convolutional neural network with a relational network (dilated DCNN+RN).

Figure 1 summarizes a novel DL‐based preoperative evaluation framework that utilizes clinical DWI tractography data to objectively predict individual children's GASE scores using our dilated DCNN+RN based on our previous works [20, 21]. First, whole‐brain tractography was generated using the methods described in second‐order integration over fiber orientation distributions (iFOD2) algorithm [28] with 2000 dynamically randomized seeding points on the gray matter/white matter interface. Secondly, whole‐brain tractography was classified into 1477 true positive tract classes, C_1‐1477_, using our previous DCNN‐based true positive tract classification [29] that could remove all possible false positive tracts such as wiggly tracked, or broken fiber tracts that are anatomically irrelevant resulting from inaccurate fiber orientation estimates. Using the resulting C_1‐1477_, we constructed true‐positive whole‐brain network, G = (Ω, S), where Ω_i_ represents a set of 724 regions of interest (ROIs), and each element S_i,j_ represents an edge quantifying the average fractional anisotropy (FA) value of the fiber tracts connecting Ω_i_ and Ω_j_, thereby representing the strength of structural connectivity between them. Thirdly, a GASE‐based ESN, g(m, n), was constructed from the G = (Ω, S) of the development set (*n* = 55). This network comprised specific connectivity edges S_m,n_ that were significantly correlated with the clinician‐assigned GASE scores of the 55 subjects, *t*_k = 1–55_. Specifically, all elements S_ij_ of the G with a Pearson's correlation *p*‐value < 0.001 after controlling for age and sex were selected as the final elements of g(m, n). Finally, the dilated DCNN+RN was trained and optimized to predict GASE score, ${\hat{t}}_{k}$, from the g(m, n) of the kth subject available in the development set. The dilated DCNN consisted of four convolutional layers, with the final two layers employing dilated kernels using a dilation factor of 2 to expand the receptive field. The subsequent RN module took as input the feature map produced by dilated DCNN and constituted an objective function (g) which had four fully connected layers, each comprising 512 units and utilizing leaky rectified linear unit (ReLU) activation with a slope of 0.2. The pairwise relational outputs of the object function were aggregated using average pooling. This pooled representation was then passed through a nonlinear function (f), which included two fully connected layers each with 512 units, leaky ReLU activation with a slope of 0.2, and 50% dropout, followed by an additional fully connected layer with 512 units and Leaky ReLU activation with a slope of 0.2. The output of the nonlinear function served as the final prediction of the dilated DCNN+RN, the predicted GASE scores, ${\hat{t}}_{k=1-55}$. The prediction performance of our dilated DCNN+RN was compared with those of several baseline models including support vector regressor (SVR) [30], linear regression model with L1 prior (LRM) [31], multiple layer regressor (MLP) [31] and other DL‐based state‐of‐the‐art models including BrainNetCNN [32] and deep residual neural network (DRNN) [18, 33] (Table S2).

All prediction models were trained and evaluated under the same experimental conditions to ensure fair performance comparison. In the development set (*n* = 55), the model evaluation was performed using leave‐one‐subject‐out cross‐validation (LOSOCV), where the data from one subject was reserved for validation, while the data from remaining subjects (i.e., *n* = 54) were used for training in each fold. To address overfitting and imbalance problems, data augmentation was applied to the g(m, n) using the synthetic minority oversampling technique for regression with Gaussian noise (SMOGN) [34], in which 6 nearest neighbors and 2% Gaussian perturbation were utilized to generate an augmentation factor of 200 for each fold of the LOSOCV, yielding *n* = 10,800 artificial instances for learning diverse patterns of g(m, n) with their corresponding GASE scores.

The generalizability of the trained dilated DCNN+RN was evaluated in the independent test set, where the G = (Ω, S) of the kth subject (i.e., *k* = 1,2,…,25) was reconstrued with the same procedure used for the development set. The g(m, n) of the kth subject was created with the same S_m,n_ identified in the development set and used to predict the GASE score using each of 55 models that were trained in the LOSOCV. An average value of these 55 predicted GASE scores was used as the final prediction of the GASE score, ${\hat{t}}_{k}$ of the kth subject. The overall reliability of the predicted GASE scores, ${\hat{t}}_{k=1-25}$ in comparison to the clinician‐assigned GASE scores, *t*_k = 1–25_ was evaluated using the following four metrics: (1) Pearson's correlation coefficient (*r*), (2) the mean absolute error (MAE), (3) the standard deviation of absolute error (SDAE), and (4) *P*_*MAE<1SD*_ the probability of MAE less than one standard deviation (SD) of our 25 subjects. Higher *r* and lower MAE indicate that the predicted scores are closer to the assigned ones. A lower SDAE indicates that the predicted scores do not have high deviation, and a larger *P*_*MAE<1SD*_ indicates that the probability of the predicted scores close to the assigned scores by a margin less than one SD of our 25 subjects is higher. Finally, to assess the extent of potential overfitting, we performed a random score learning experiment by repeating both the training and testing procedures of the dilated DCNN+RN with pseudo GASE scores that were randomly assigned to individual subjects in the range of 7–49. It was assumed that if the *r* values resulted from this experiment were not statistically significant, the impact of overfitting at the given sample size would be minimal.

### 2.6 Imaging‐Based Quantification of Neurocognitive Risk Using Hazard Ratio Analysis

Univariate and multivariate Cox proportional hazards models [23] were employed for DRE patients to estimate the HR values and their corresponding 95% confidence intervals (CIs) for the evaluation of the risk associated with progressive neurocognitive impairment in class I: general ability, class II: verbal ability, and class II: non‐verbal ability (outcome: HR value) using different types of epilepsy severity indicators that are available at the time of preoperative evaluation (predictor: GASE score, lesion type/location visible in MRI = [focal cortical dysplasia (FCD) in the temporal lobe, FCD in extra‐temporal regions, other temporal lobe lesions including tumor, hippocampal sclerosis (HS), and encephalitis (Ence), other extra‐temporal lesions including tumor, HS, and Ence in extra‐temporal regions, no lesion visible in MRI]).

Briefly, the outcome, defined as impairment risk in class I: general cognitive ability, class II: verbal ability, and class III: non‐verbal ability, was considered as an event of interest (i.e., high risk: score ≤ 3 vs. low risk: score > 3). Each model evaluates the effect of the model indicators on the event time as their HR values, which reflect the individual contribution of each indicator to the overall risk. To estimate the HR values in the univallate model, the assigned GASE score, *t*_k_ was considered in model 1, while the predicted GASE score, ${\hat{t}}_{k}$ was considered in model 2. Similarly, model 1 of the multivariate analysis incorporated the assigned GASE score, *t*_k_ in conjunction with the additional MRI indicator, lesion type/location visible in MRI. Model 2 included the predicted GASE score, ${\hat{t}}_{k}$ alongside the same MRI indicator to assess its independent associations.

## 3 Results

### 3.1 Construction of GASE‐Based ESN

**FIGURE 2:**
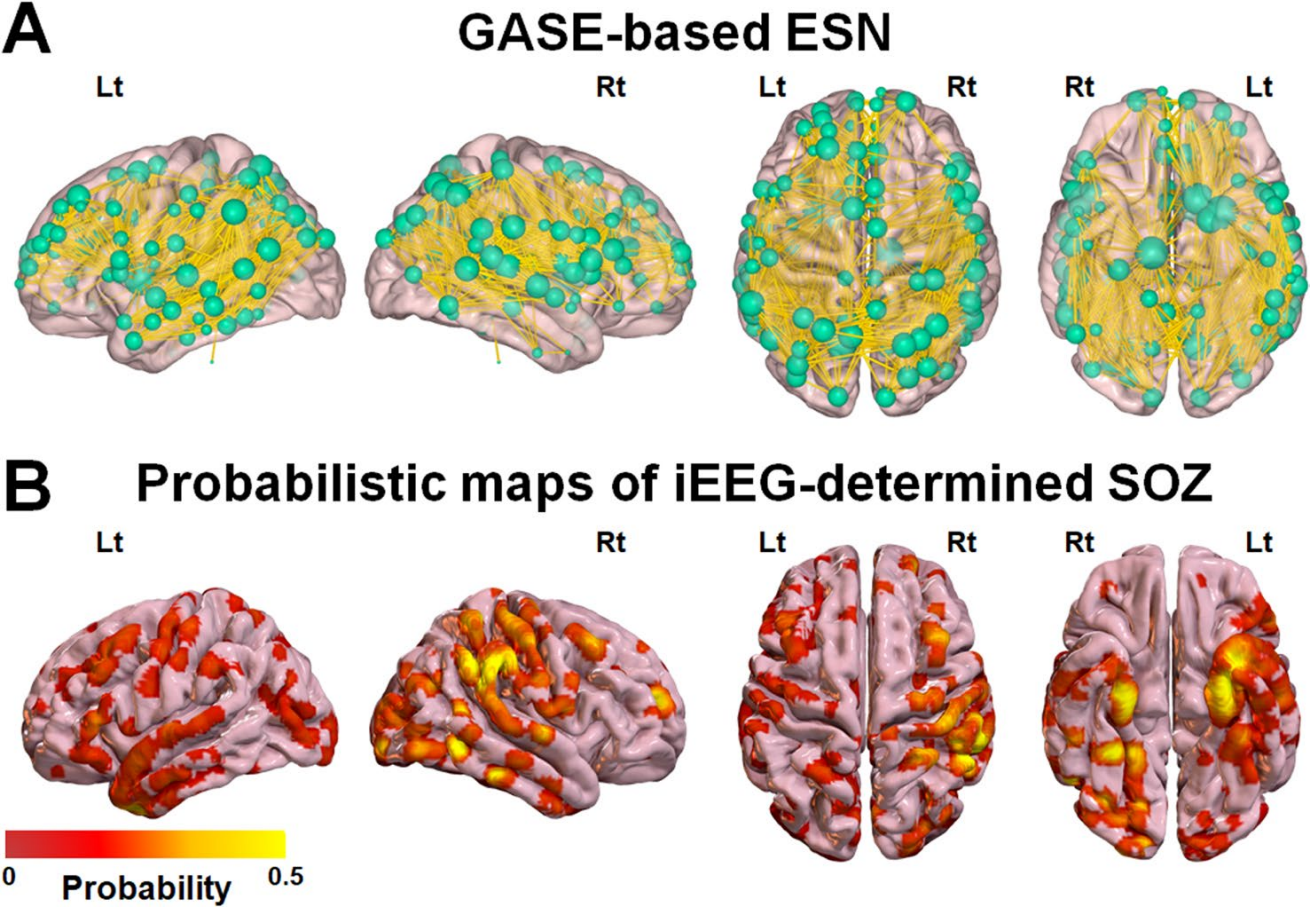
(A) GASE‐based ESN, g(m, n), derived from the correlation between the edge strengths of true positive whole‐brain network, G = (Ω, S), and the clinician‐assigned GASE scores of the development set (*n* = 35 DRE patients). A total of 115 ROIs were identified as key nodes of g(m, n) for m(or n) = 1,2, …,115 at a significance threshold of correlation *p*‐value < 0.001, after controlling age and sex. The radius of each green sphere indicates the sum of edge strengths, S_m,n_ (i.e., yellow‐colored streamlines) connected with each node, Ω_m_. (B) Overlapping iEEG‐determined SOZ electrodes in a standard freesurfer average brain template. The color bar indicates the inter‐subject likelihood of SOZ electrode (*n* = 35 DRE patients in the development set) overlap at each vertex on the 3D‐brain template. It is notable that smaller spheres in (A) correspond to higher probabilities of SOZ overlap, suggesting a greater impact of GASE score on edge strengths at the group level.

Figure 2A shows a representative example of the GASE‐based ESN, g(m, n), derived from the correlation between the edge strengths of the true positive whole‐brain network, G = (Ω, S), and the measured GASE scores of the development set (*n* = 55). A total of 115 ROIs were identified as key regions of g(m, n) for m (or n) = 1,2,…,115 at a significance threshold of correlation *p*‐value < 0.001, after controlling for age and sex. The anatomical locations of these regions are listed in Table S3. Notably, most iEEG‐determined seizure onset zones (SOZ), defined by overlapping SOZ electrodes in a standard freesurfer average brain template [35], were included in the g(m, n) of 36 DRE patients available in the development set (Figure 2B), suggesting that the edge strengths of SOZ nodes were significantly reduced by the seizure activities at the group level, resulting in higher GASE scores. Although our group‐level analysis indicates that regions associated with the SOZ sites may significantly contribute to overall epilepsy severity, it is important to clarify that the GAN‐based ESN is not designed for, nor capable of, localizing SOZ sites at the individual patient level, and therefore cannot be used to guide clinical SOZ localization on a case‐by‐case basis.

### 3.2 Prediction of Epilepsy Severity Using the Proposed Imaging Biomarkers

Table 1 presents overall performance of the dilated DCNN + RN and other models in predicting GASE scores from 25 subjects of the independent test set. Clearly, the dilated DCNN + RN outperformed all other models to predict the GASE scores, ${\hat{t}}_{k}$, from the k^th^ patient's GASE‐based ESN, g(m, n), achieving the highest Pearson's correlation (*r* = 0.83) and probability of MAE (P_MAE<1SD_ = 0.92) as well as the lowest MAE (MAE = 4.98) and SDAE (SDAE = 6.40). When compared with the latest state‐of‐the art DL model, DRNN, our dilated DCNN+RN led to an average improvement of 2%, 28%, 35%, and 27% in *r*, P_MAE<1SD_, MAE, SDAE, respectively, suggesting systematic dilation operator of the g(m, n) could effectively capture the complex, non‐local connectivity patterns of the g(m, n), which may play a critical role in enhancing the imaging‐based prediction of GASE scores across individual DRE patients. Despite the relatively small sample size (*n* = 80), the trained dilated DCNN+RN model yielded outstanding predictive performance comparable on both the development set (i.e., *r* = 0.92, MAE = 2.39, SDAE = 3.96; random score *r* = −0.255, MAE = 11.87, SDAE = 14.62) and the independent test set (i.e., *r* = 0.83, MAE = 4.98, SDAE = 6.40; random score *r* = 0.00, MAE = 11.23, SDAE = 13.05). This consistency suggests that the use of SMOGN for data augmentation was effective in mitigating overfitting to a specific training set.

**TABLE 1:** Performance comparison of the dilated DCNN+RN with other models for predicting the global assessment of the severity of epilepsy (GASE) scale scores. The Pearson's correlation coefficient (*r*) and its *p*‐value, mean absolute error (MAE), standard deviation of absolute error (SDAE), and the probability of MAE less than one standard deviation of the study cohort (*P*_MAE<1SD_) were evaluated in the independent test set (*n* = 25).

Regression model	*r*	*p*‐value	MAE	SDAE	*P*_MAE<1SD_
SVR	0.16	0.441	10.15	11.12	0.52
Lasso	0.67	< 0.001	10.26	11.28	0.56
MLR	0.71	< 0.001	7.95	9.29	0.84
BrainNetCNN	0.74	< 0.001	6.76	8.40	0.88
DRNN	0.81	< 0.001	7.61	8.78	0.72
Dilated DCNN + RN (assigned GASE score)	0.83	< 0.001	4.98	6.40	0.92
Dilated DCNN + RN (random GASE score)	0.00	0.996	11.23	13.05	0.56

**FIGURE 3:**
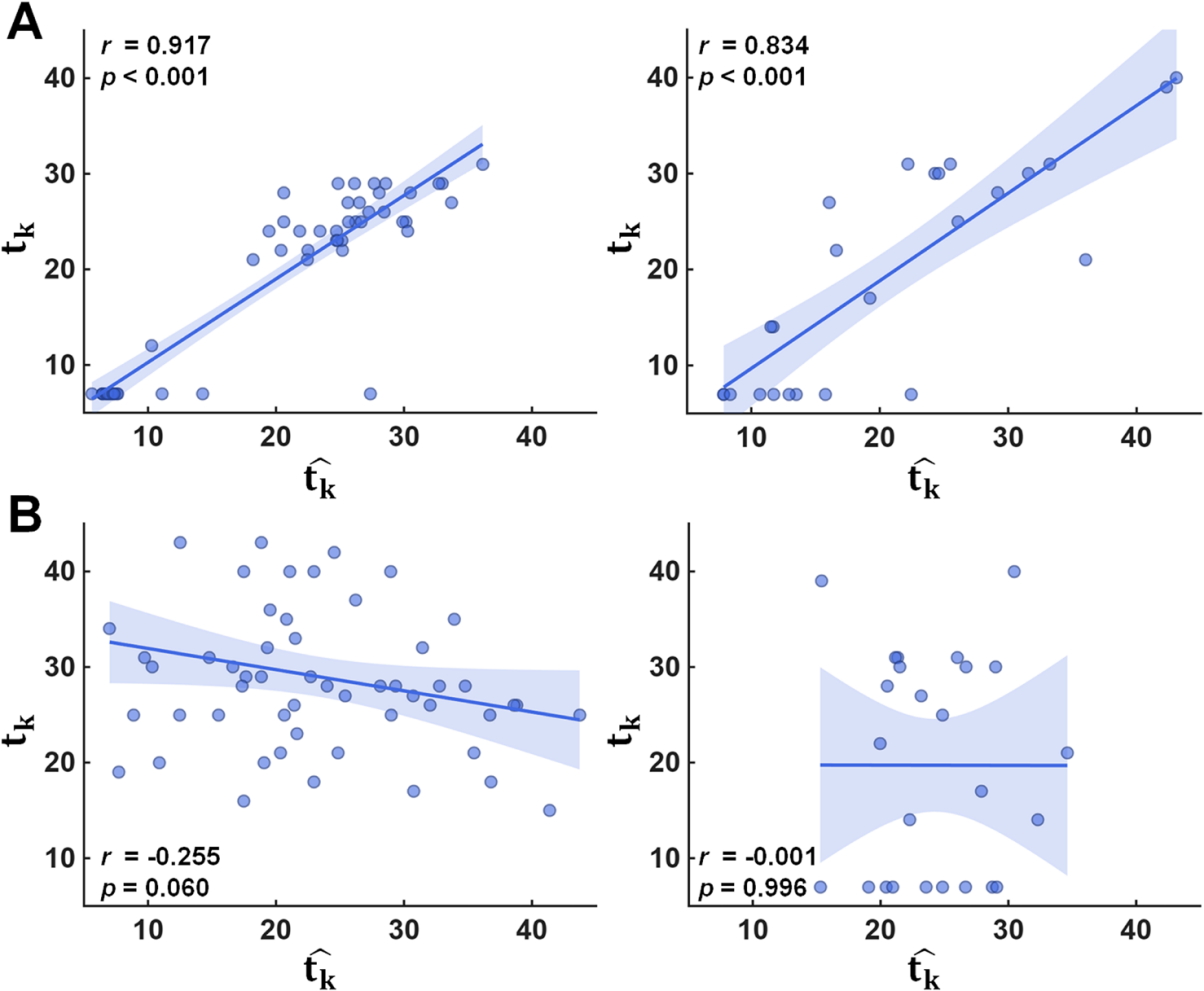
(A) Results from the original score learning experiment. (B) Results from the random score learning experiment to assess potential overfitting. Using the development set, two dilated DCNN+RN models were separately trained to predict the proposed biomarker, ${\hat{t}}_{k}$, utilizing the g(m, n) of the kth DRE patient with (A) the clinician‐assigned GASE score, *t*_k_ and (B) the pseudo GASE score, *t*_k_ randomly assigned in the range of 7–49. In each experiment, the trained model was used to predict the proposed biomarker, ${\hat{t}}_{k}$ from the g(m, n) of the kth DRE patient in both development set (left) and independent test set (right). The shade represents the 95% confidence interval.

The outstanding model fit along with 95% confidence interval was found in the prediction of the dilated DCNN+RN (Figure 3). Compared to the random score learning experiment (Figure 3B), the original score learning experiment (Figure 3A) produced substantially higher coefficient correlation between the clinician‐assigned score, *t*_k_ and the predicted score, ${\hat{t}}_{k}$ across both the development and test datasets. This indicates that the dilated DCNN+RN was able to effectively learn detailed features of the g(m, n), enabling accurate prediction of the assigned scores with minimal overfitting, despite the limited sample size of the development set (*n* = 55). The result of this comparison suggests high potential of the proposed biomarker, ${\hat{t}}_{k}$ to augment or substitute components of time‐consuming epilepsy severity assessments in clinical cases with limited data availability, even where information is limited and unreliable for individual DRE patients.

### 3.3 Quantification of Neurocognitive Risk Using the Predicted GASE Score

**FIGURE 4:**
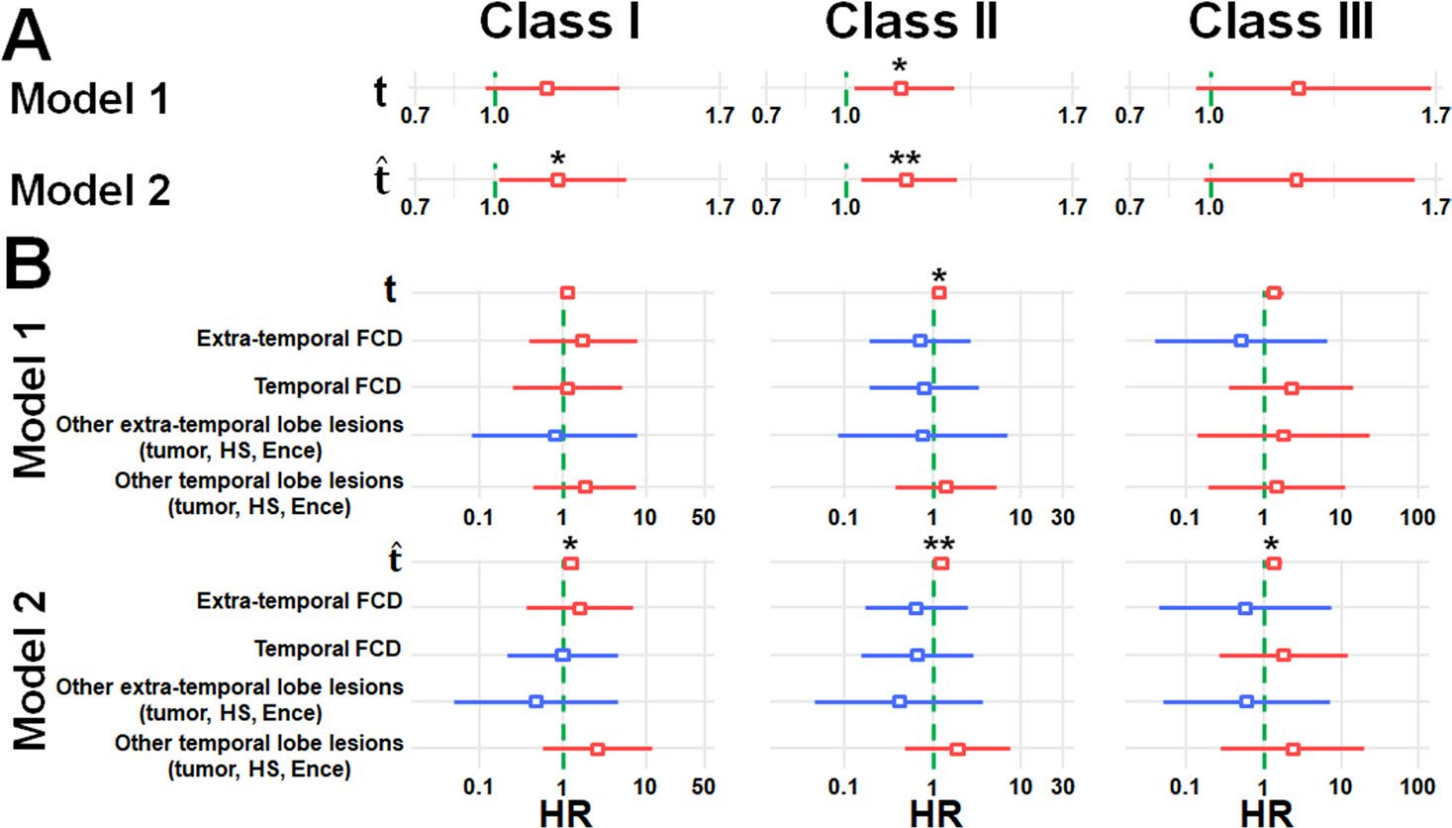
Results of (A) univariate and (B) multivariate Cox proportional hazards models using two configurations: model 1 which incorporates the clinician‐assigned GASE score, *t*, and model 2 which utilizes the predicted GASE score, $\hat{t}$, to estimate the HR values for three neurocognitive impairment risks, class I: general cognitive ability, class II: verbal ability, and class III: non‐verbal ability. Boxes and horizontal bars indicate the estimated HR values and their 95% confidence intervals, respectively. Red and blue represent increased and decreased risk of neurocognitive impairment, respectively. *, **, and *** indicate the *p*‐values of the HR estimates < 0.05, 0.01, and 0.001, respectively.

Figure 4, Table S4, based on three subsets of full study cohort (*n* = 36, 37, and 32 DRE patients for class I, class II, and class III, respectively), present a comparison between the performance of univariate and multivariate Cox proportional hazards models using two configurations: model 1 which incorporates the clinician‐assigned GASE score, t_k_ and model 2 which utilizes the predicted GASE score, ${\hat{t}}_{k}$. This comparison was intended to demonstrate the superior robustness of the predicted GASE score in quantifying the neurocognitive risks of individual DRE patients, as reflected by the HR values of individual predictors that were used to assess the risk of neurocognitive impairment in class I: general cognitive ability, class II: verbal ability, and class III: non‐verbal ability. In the univariate model, the predicted GASE score in model 2 showed a stronger association with the hazard of the outcome (HR/CI/*p*‐value = 1.19/1.02‐1.39/0.028 for class I; 1.18/1.05‐1.33/0.006 for class II; 1.26/0.99‐1.61/0.065 for class III) compared to the assigned GASE score in model 1 (1.15/0.97‐1.35/0.103 for class I; 1.16/1.02‐1.31/0.022 for class II; 1.25/0.96–1.63/0.100 for class III). Furthermore, model 2 demonstrated superior model performance, with a lower Akaike Information Criterion (AIC = 76.69 vs. 79.33 for class I; 87.19 vs. 89.83 for class II; 37.20 vs. 37.98 for class III) and a higher bias‐corrected Harrell's C‐index (0.719 vs. 0.618 for class I; 0.717 vs. 0.641 for class II; 0.637 vs. 0.653 for class III), indicating better model fit and discrimination. These findings suggest that model 2 provides a more informative predictor of risk in the given cohort. Furthermore, the multivariate model found that model 2 using the predicted GASE score with the additional MRI indicator provided more significant HR values (HR/CI/*p*‐value = 1.23/1.03‐1.48/0.022 for class I; 1.22/1.07‐1.40/0.003 for class II; 1.33/1.02‐1.74/0.036 for class III) when compared with model 1 using the assigned GASE score with the same additional MRI indicator (1.12/0.93‐1.35/0.215 for class I; 1.17/1.02–1.34/0.025 for class II; 1.34/0.99‐1.81/0.057 for class III). Also, model 2 exhibited superior performance, as evidenced by the lower AIC (81.20 vs. 86.20 for class I; 92.26 vs. 96.85 for class II; 43.15 vs. 44.14 for class III) and the higher bias‐corrected Harrell's C‐index (0.686 vs. 0.518 for class I; 0.685 vs. 0.582 for class II; 0.626 vs. 0.614 for class III), indicating improved model fit and discriminatory ability. This suggested that the predicted GASE score, ${\hat{t}}_{k}$ provides a more adjusted and accurate estimate of epilepsy severity contributing to the overall risk.

Based on three subsets of the full study cohort (*n* = 36, 37, and 32 DRE patients for class I, class II, and class III, respectively), Table 2 demonstrates that both model 1 and model 2 could demonstrate statistically significant differences between the high‐risk group and the low‐risk group, as assessed by Welch's *t*‐test (*p*‐values of model 1/2: 0.008/0.002, 0.042/0.014, 0.017/0.008 for class I, II, III). Effect size analysis using Hedges' g indicated a large difference for model 1 (g = −0.93, −0.70, −0.81 for class I, II, III) and a very large difference for model 2 (*g* = −1.10, −0.86, −0.87 for class I, II, III). These findings suggest that while both models 1 and 2 are effective in distinguishing between the groups, model 2 shows a stronger discriminatory capacity, both in terms of statistical significance and effect magnitude. More significant separation and higher effect size between the estimated HR values of the two groups demonstrate that the predicted GASE score outperforms the assigned GASE score to quantify the overall risk of neurocognitive impairment across individual DRE patients.

**TABLE 2:** Comparison of log‐hazard ratios obtained from multivariate Cox proportional hazards model analyses and their statistical significance levels between two groups of specific neurocognitive impairments: low and high‐risk groups in class I: general cognitive ability (*n* = 19 and 17), class II: verbal ability (*n* = 18 and 19), and class III: non‐verbal ability (*n* = 22 and 10).

Model type	Group	Mean ± SD	*t*(df)	*p*‐value	Hedges' g (95% CI)
1	Class I (Low)	−0.04 ± 0.61	−2.81 (33.35)	0.008**	−0.93 (−1.64, −0.21)
	Class I (High)	0.47 ± 0.47			
2	Class I (Low)	−0.36 ± 1.26	−3.38 (30.06)	0.002**	−1.10 (−1.82, −0.37)
	Class I (High)	0.80 ± 0.76			
1	Class II (Low)	−0.34 ± 0.88	−2.12 (31.98)	0.042*	−0.70 (−1.39, −0.01)
	Class II (High)	0.21 ± 0.68			
2	Class II (Low)	−0.62 ± 1.30	−2.60 (31.67)	0.014*	−0.86 (−1.56, −0.16)
	Class II (High)	0.37 ± 0.98			
1	Class III (Low)	−0.12 ± 1.40	−2.54 (27.00)	0.017*	−0.81 (−1.62, −0.01)
	Class III (High)	0.91 ± 0.86			
2	Class III (Low)	−0.26 ± 1.68	−2.84 (29.01)	0.008**	−0.87 (−1.68, −0.06)
	Class III (High)	1.04 ± 0.90			

*Note:* * and ** represent *p*‐value < 0.05 and 0.01, respectively.

Abbreviations: CI, confidence interval; Low/High, low−/high‐risk of neurocognitive impairment occurrence based on the assumption of neuropsychological standard score (Low: score > 3 vs. High: score ≤ 3); *t*(df), *t*‐statistics(degree of freedom).

## 4 Discussion

The present study demonstrated the feasibility of our novel DWI tractography‐based ESN biomarkers to quantify the degree of epilepsy severity and predict the progressing risk of neurocognitive impairment. The assessment of epilepsy severity using the current GASE score remains largely subjective and continues to be one of the standard approaches for guiding the prevention of neurocognitive impairment. However, it does not directly capture the impact of epilepsy risk on neurocognition. This study fills this gap by incorporating state‐of‐the art deep learning technology with preoperative DWI tractography data to create a new epilepsy severity biomarker, predicted GASE score ($\hat{t}$), that could accurately predict the HR values using the Cox proportional hazards risk model to differentiate progressing neurocognitive risks in class I: general cognitive ability, class II: verbal ability, and class III: non‐verbal ability.

**FIGURE 5:**
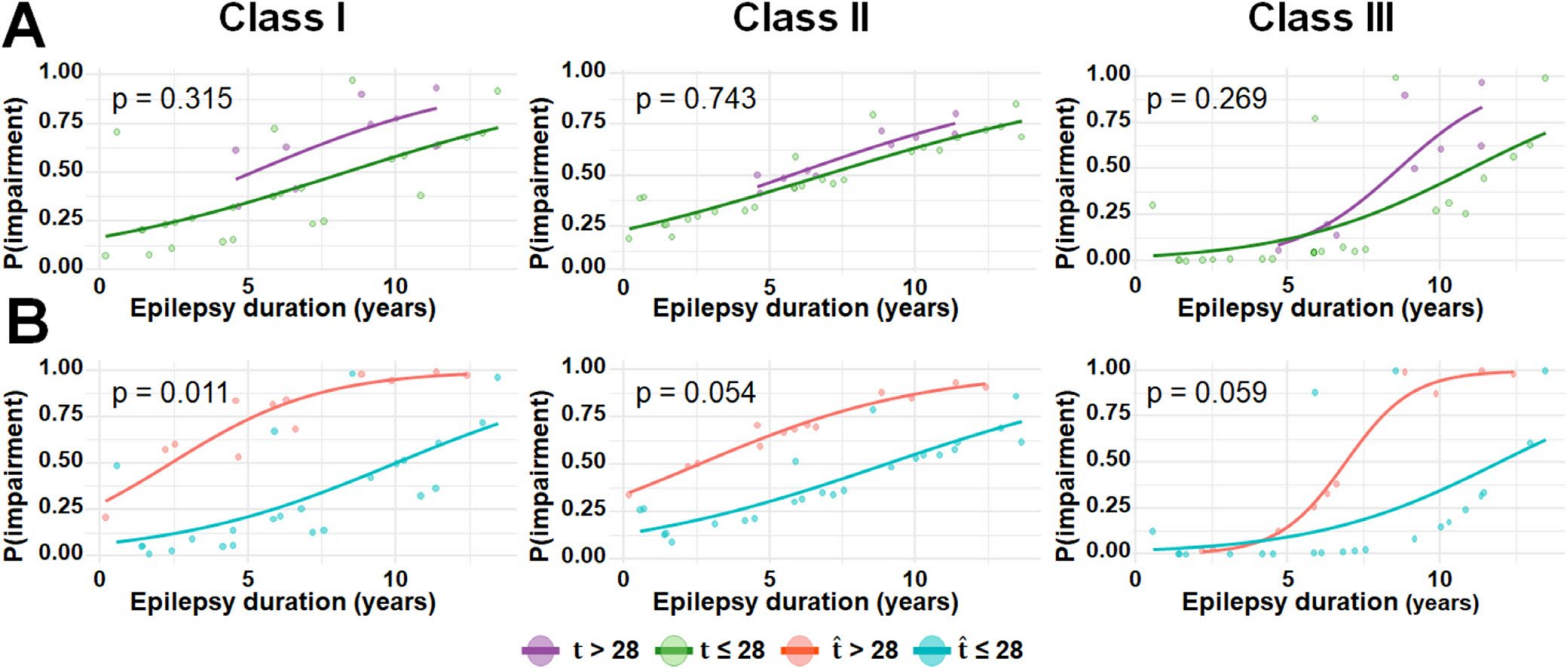
(A) Logistic regression time analysis using the clinician‐assigned GASE score, *t*, and (B) Logistic regression time analysis using the predicted GASE score, $\hat{t}$. Each analysis estimated probability that individual DRE patients exhibited neurocognitive impairment for a given epilepsy duration, time in years. In each of three classes, the logistic regression test yielded a more significant *p*‐value when based on the predicted GASE score, $\hat{t}$. The median value of the 51 clinician‐GASE scores (i.e., 28) was used as a threshold to determine the event occurrence (i.e., neurocognitive impairment being presented).

To our best knowledge, the present study is the first to perform the proof‐of‐concept for the importance of DWI tractography‐based epilepsy severity biomarkers to mine deep learning features of white matter abnormalities directly measured at specific brain networks of DRE children and use those features to predict the risk of neurocognitive impairment. The findings of this study may greatly improve epilepsy care by offering a reliable, objective, and comprehensive picture of how severe epilepsy impacts the brain, supporting better diagnosis, monitoring, and treatment strategies to prevent long‐term neurocognitive impairments. For instance, epilepsy duration (i.e., interval between seizure onset age and preoperative neuropsychological assessment age) could serve as the time‐to‐event variable, with “impairment being presented” treated as an event of interest (e.g., High: score ≤ 3 vs. Low: score > 3). Using the predicted GASE score, $\hat{t}$, as a predictor for the event, specific time thresholds of epilepsy duration (e.g., 2.5 years for class I and II, 7 years for class III) could be found for intervention timing aiming at preventing neurocognitive impairments, where the probability that individual subjects exhibited neurocognitive impairment for a given epilepsy duration was evaluated by performing the conventional logistic regression analysis [36]—it is clear that the subjective nature of the clinician‐assigned GASE score could not find these critical thresholds, as it failed to clearly distinguish between the likelihood plots of the two groups (e.g., *p*‐values > 0.2, Figure 5). Although exploratory and cross‐sectional in nature, this temporal information provided by the predicted GASE score may play a critical role in guiding the optimal timing of surgical intervention for DRE children to minimize the risk of long‐term neurocognitive consequences resulting from the prolonged seizures.

Brain development is most dynamic in the first few years of life with cortical expansion and myelination of white matter tracts [37]. This includes the development of both the cortex and white matter tracts/networks associated with neurocognition including speech and language. Quantitative MRI studies [38, 39] have demonstrated consistent, rapid macrostructural white matter development over the first 3 years of life. Previous DWI studies [40, 41] also reported dynamic changes attributed to continued axonal packing, white matter maturation, synaptogenesis, synaptic pruning, and remodeling during later childhood. Since rapid synaptogenesis peaks at 24–36 months of age and subsequently starts pruning and maturation [42], repeated epileptic activity during this critical period can lead to a condition known as epileptic encephalopathy [43, 44], in which the epileptic process itself may become a major contributor to brain changes underlying neurocognitive and behavioral dysfunction. For instance, blurring of the gray‐white matter interface is often visible on MRI and considered as common hallmarks in temporal lobe epilepsy and neocortical epilepsies associated with developmental malformations [45]. Also, it was reported that abnormal myelination develops early because of the repeated seizures [46, 47], altering diffusivity properties near epileptic foci and their axonal pathways involved in the propagation of seizures. Other DWI studies [48, 49, 50] have shown that children with more severe epilepsy often exhibit reduced FA in whole‐brain. Since white matter abnormalities correlate with neurocognitive impairments [48], which are often associated with more severe epilepsy, the assessment of white matter integrity may provide a valuable insight into both the biological severity of epilepsy and its potential functional consequences, helping clinicians tailor management and predict outcomes more effectively. Future studies could confirm that the proposed biomarker, $\hat{t}$ could be used for personalized medicine, tailoring therapy based on imaging evidence of seizure impact by facilitating the stratification of DRE patients who are at elevated risk for neurocognitive impairment, thereby prioritizing those who may most likely benefit from early surgical evaluation aimed at reducing epilepsy severity.

The present study has several limitations. First, although the GASE score has been widely utilized for assessing epilepsy severity, more advanced assessment tools such as the pediatric epilepsy severity scale, Chalfont seizure severity scale, and national hospital seizure severity scale were not considered. Thus, the reported findings should be carefully considered as a proof‐of‐concept supporting that the advanced DL‐based DWI tractography analysis holds potential to offer an objective, imaging‐based epilepsy severity marker that may help address the current shortcoming of epilepsy severity assessment tools that are inherently subjective and particularly challenging to evaluate in pediatric populations. Secondly, the limited sample size of this study was primarily attributable to missing preoperative tractography data for determining the presence of neurocognitive impairment. Due to the limited sample size, the framework of LOSOCV was used to train and validate the dilated DCNN+RN in the development set, which may produce an overoptimistic view of generalizability by implicitly learning common patterns across subjects. While the trained models could effectively capture inter‐subject generalizability, they may overlook intra‐subject variability, which may be critical for longitudinal measurements. In addition, the findings derived from the HR models using predicted GASE scores were based on only subsets of the full cohort as disclosed in the subsection: Evaluation of neurocognitive impairment, as age‐appropriate assessments of global, verbal, and non‐verbal IQ could only be administered and completed within these specific subsets. Consequently, this unavoidable selection could introduce a potential bias, which may limit the generalizability of the HR models' performance to the entire study cohort. Thirdly, due to the retrospective nature of this study, it was not feasible to assess the efficacy of the proposed epilepsy severity marker using longitudinal measurements. Consequently, the reported risk estimates were inevitably derived from a cross‐sectional analysis and should be interpreted at the population level of the study cohort. Within such study designs, the efficacy of the proposed biomarker should be compared with those of other modality markers such as interictal EEG measures and will be tested with other institutional data, which is crucial to validate the generalizability of the proposed biomarkers to other data sources.

## Author Contributions

Jeong‐Won Jeong: (1) conception and design of the study, (2) acquisition and analysis of data, or (3) drafting a significant portion of the manuscript or figure. Min‐Hee Lee: (1) analysis of data, (2) drafting a significant portion of the manuscript or figure. Hiroshi Uda: (1) analysis of data. Yoon Ho Hwang: (1) analysis of data. Michael Behen: (1) analysis of data, (2) drafting a significant portion of the manuscript or figure. Aimee Luat: (1) analysis of data, (2) drafting a significant portion of the manuscript or figure. Csaba Juhász: (1) drafting a significant portion of the manuscript or figure. Eishi Asano: (1) acquisition and analysis of data, (2) drafting a significant portion of the manuscript or figure.

## Conflicts of Interest

The authors declare no conflicts of interest.

## Supporting information


**Table S1:** Clinical variables of the 51 study subjects.
**Table S2:** Baseline models and other DL‐based models used for the comparison.
**Table S3:** Brain regions that significantly correlated with GASE score and comprise the GASE‐based SLN.
**Table S4:** Univariate and multivariate Cox proportional hazards model estimating the hazard ratio (HR) of cognitive impairment in class I: general cognitive ability, class II: verbal ability, and class III: non‐verbal ability.

## Data Availability

We have made the open‐source implementation of the dilated CNN+RN, as well as the associated trained models and sample datasets, publicly available for research and education purposes (https://github.com/wsutil/dilated‐CNN‐RN‐GASE). All data utilized in this study is also available from the corresponding author upon reasonable request.
