# Supplementary material for: Diffusion Tractography Biomarker for Epilepsy Severity in Children With Drug‐Resistant Epilepsy
Source: Ann Clin Transl Neurol. 2025 Oct 8;13(2):343–53. doi: 10.1002/acn3.70217 (PMC12883680; doi:10.1002/acn3.70217)
Supplement: Supplementary file 1 — Table S1: Clinical variables of the 51 study subjects. Table S2: Baseline models and other DL‐based models used for the comparison. Table S3: Brain regions that significantly correlated with GASE score and comprise the GASE‐based SLN. Table S4: Univariate and multivariate Cox proportional hazards model estimating the hazard ratio (HR) of cognitive impairment in class I: general cognitive ability, class II: verbal ability, and class III: non‐verbal ability. [file ACN3-13-343-s001.docx]

**Supplementary table 1.** Clinical variables of the 51 study subjects.

| Case No. | Age  (years) | Sex | Surgery  outcome | Surgery side | seizure onset age | epilepsy duration | seizure frequency | the number of AED |
| --- | --- | --- | --- | --- | --- | --- | --- | --- |
| 1 | 10.3 | B | 1 | Lt | 4.0 | 6.3 | monthly | 3 |
| 2 | 12.1 | G | 2 | Lt | 9.0 | 3.1 | weekly | 2 |
| 3 | 9.9 | G | 3 | Rt | 4.0 | 5.9 | monthly | 2 |
| 4 | 14.1 | B | 1 | Lt | 8.0 | 6.1 | monthly | 2 |
| 5 | 4.9 | B | 1 | Lt | 2.5 | 2.4 | daily | 3 |
| 6 | 13.8 | G | 5 | Rt | 0.3 | 13.5 | daily | 3 |
| 7 | 9.2 | G | 1 | Lt | 7.0 | 2.2 | weekly | 2 |
| 8 | 11.5 | G | 1 | Lt | 0.1 | 11.4 | daily | 3 |
| 9 | 16.0 | B | 4 | Lt | 6.0 | 10.0 | monthly | 3 |
| 10 | 17.2 | B | 1 | Rt | 10.0 | 7.2 | monthly | 2 |
| 11 | 9.4 | G | 1 | Lt | 8.0 | 1.4 | monthly | 1 |
| 12 | 12.8 | B | 1 | Lt | 7.0 | 5.8 | daily | 2 |
| 13 | 11.6 | G | 1 | Lt | 0.2 | 11.4 | weekly | 2 |
| 14 | 6.5 | G | 1 | Lt | 1.0 | 5.5 | monthly | 2 |
| 15 | 12.4 | G | 1 | Lt | 11.0 | 1.4 | monthly | 1 |
| 16 | 10.9 | G | 3 | Rt | 5.0 | 5.9 | daily | 2 |
| 17 | 11.6 | B | 1 | Rt | 1.1 | 10.5 | daily | 2 |
| 18 | 11.7 | G | 1 | Rt | 7.0 | 4.7 | monthly | 2 |
| 19 | 10.6 | B | 1 | Rt | 0.6 | 10.0 | weekly | 3 |
| 20 | 16.1 | B | 3 | Lt | 12.0 | 4.1 | weekly | 2 |
| 21 | 13.8 | B | 1 | Rt | 3.0 | 10.8 | monthly | 3 |
| 22 | 8.1 | B | 5 | Rt | 0.5 | 7.6 | daily | 2 |
| 23 | 15.7 | G | 1 | Lt | 13.0 | 2.7 | weekly | 1 |
| 24 | 5.6 | G | 5 | Lt | 5.0 | 0.6 | daily | 3 |
| 25 | 17.0 | B | 1 | Lt | 4.0 | 12.9 | monthly | 3 |
| 26 | 8.5 | B | 3 | Rt | 0.0 | 8.5 | daily | 2 |
| 27 | 13.9 | G | 3 | Rt | 4.0 | 9.9 | weekly | 3 |
| 28 | 5.9 | G | 1 | Lt | 1.4 | 4.5 | daily | 2 |
| 29 | 16.5 | B | 1 | Rt | 12.0 | 4.5 | yearly | 2 |
| 30 | 13.8 | G | 1 | Rt | 5.0 | 8.8 | monthly | 3 |
| 31 | 16.7 | B | 4 | Rt | 0.0 | 16.7 | daily | 2 |
| 32 | 14.5 | G | 2 | Lt | 12.0 | 2.5 | daily | 3 |
| 33 | 11.8 | B | 1 | Rt | 3.0 | 8.8 | daily | 3 |
| 34 | 13.4 | G | 5 | Lt | 1.0 | 12.4 | weekly | 3 |
| 35 | 11.3 | G | 1 | Lt | 1.0 | 10.3 | weekly | 2 |
| 36 | 13.6 | B | 3 | Lt | 7.0 | 6.6 | weekly | 2 |
| 37 | 10.7 | B | 1 | Rt | 1.5 | 9.2 | daily | 2 |
| 38 | 5.5 | G | 1 | Lt | 1.0 | 4.5 | daily | 3 |
| 39 | 6.2 | B | 5 | Rt | 6.0 | 0.2 | monthly | 2 |
| 40 | 15.8 | G | 3 | Rt | 5.0 | 10.8 | monthly | 2 |
| 41 | 10.6 | B | 3 | Rt | 9.0 | 1.6 | weekly | 2 |
| 42 | 8.2 | B | 1 | Lt | 6.0 | 2.2 | monthly | 2 |
| 43 | 10.3 | B | 1 | Lt | 8.0 | 2.2 | weekly | 3 |
| 44 | 16.5 | G | 3 | Rt | 12.0 | 4.5 | monthly | 2 |
| 45 | 12.8 | G | 1 | Rt | 5.0 | 7.8 | weekly | 3 |
| 46 | 7.5 | G | 1 | Rt | 2.0 | 5.5 | monthly | 3 |
| 47 | 13.4 | G | 3 | Lt | 2.0 | 11.4 | weekly | 1 |
| 48 | 13.8 | B | 4 | Rt | 7.0 | 6.8 | yearly | 1 |
| 49 | 16.6 | B | 4 | Rt | 12.0 | 4.6 | yearly | 1 |
| 50 | 13.9 | G | 4 | Lt | 0.3 | 13.6 | yearly | 3 |
| 51 | 7.7 | G | 1 | Rt | 7.0 | 0.7 | monthly | 1 |

B, boy; G, girl; Rt, right; Lt, left;

**Supplementary table 2.** Baseline models and other DL-based models used for the comparison.

| **Category** | **Model** | **Description** |
| --- | --- | --- |
| **Baseline model** | Support vector regressor (SVR) | Epsilon-Support Vector Regressor with a linear kernel with regularization parameter C set to 1.0 and epsilon set to 0.1. |
|  | Linear Regression Model with L1 prior (Lasso) | Linear model trained with L1 prior as the regularizer with the regularization parameter set to 1.0. |
|  | Multi-Layer Regressor (MLR) | MLR constituted a four-layer fully connected network with 512 units in the first 2 layers with 50% dropout followed by 256 units in the third layer and a single linear unit in the final layer for prediction of GASE scale scores. |
| **DL-based model** | BrainNetCNN | The BrainNetCNN consisted of two main modules. Two Edge-to-Edge (E2E) convolutional layers and an Edge-to-Node (E2N) and Node-to-Graph (N2G) transformation pipeline. Briefly, two consecutive E2E layers were applied using cross-shaped convolutional filters (2 × 115), designed to capture edge-level interactions within the GASE scale modular network. Each E2E layer employed 32 filters, followed by Leaky ReLU activations with a slope of 0.33. The E2N layer utilized 32 cross-shaped filters with dimensions equal to the input connectivity matrix (i.e., (115 × 1) × (1 × 115)), strided along the diagonal to determine edge contributions to connected nodes. This was followed by an N2G transformation, 50% dropout, and a Leaky ReLU activation with a slope of 0.33. The resulting graph-level features were passed through two fully connected networks with 128 and 30 units, respectively, each followed by Leaky ReLU activations. The final output was a single linear unit for regression. |
|  | Deep residual neural network (DRNN) | The DRNN consisted of an initial convolutional layer and attention module, followed by max pooling. This was followed by three repeated modules, each comprising two residual blocks, a bottleneck branch block for auxiliary supervision, adaptive average pooling, and an auxiliary fully connected layer. After the repeated modules, the network included an additional residual block, global average pooling, and three fully connected layers with ReLU activation, and 10% dropout, which were applied for regression tasks. |

**Supplementary table 3.** Brain regions that significantly correlated with GASE score and comprise the GASE-based SLN.

| **No.** | **Anatomical regions correlated with GASE score** | **No.** | **Anatomical regions correlated with GASE score** |
| --- | --- | --- | --- |
| 1 | Left superior temporal pole F | 59 | Left posterior long insular gyrus B |
| 2 | Left superior middle temporal pole D | 60 | Right inferior middle temporal pole D |
| 3 | Left superior temporal gyrus B | 61 | Right superior temporal gyrus A |
| 4 | Left superior temporal gyrus D | 62 | Right superior temporal gyrus B |
| 5 | Left superior middle temporal gyrus A | 63 | Right superior temporal gyrus C |
| 6 | Left superior middle temporal gyrus B | 67 | Right superior temporal gyrus E |
| 7 | Left superior middle temporal gyrus D | 65 | Right superior temporal gyrus F |
| 8 | Left superior middle temporal gyrus F | 66 | Right superior temporal gyrus G |
| 9 | Left inferior middle temporal gyrus C | 67 | Right superior middle temporal gyrus F |
| 10 | Left inferior middle temporal gyrus D | 68 | Right lateral inferior temporal gyrus F |
| 11 | Left inferior middle temporal gyrus F | 69 | Right basal inferior temporal gyrus A |
| 12 | Left lateral inferior temporal gyrus C | 70 | Right superior occipital gyrus A |
| 13 | Left basal inferior temporal gyrus B | 71 | Right superior occipital gyrus E |
| 14 | Left basal inferior temporal gyrus D | 72 | Right superior middle occipital gyrus B |
| 15 | Left basal inferior temporal gyrus E | 73 | Right inferior middle occipital gyrus J |
| 16 | Left superior middle occipital gyrus B | 74 | Right superior angular gyrus A |
| 17 | Left superior angular gyrus A | 75 | Right supramarginal gyrus 1 D |
| 18 | Left inferior angular gyrus A | 76 | Right supramarginal gyrus 3 C |
| 19 | Left inferior angular gyrus D | 77 | Right supramarginal gyrus 4 A |
| 20 | Left supramarginal gyrus 1 C | 78 | Right superior parietal 1 B |
| 21 | Left supramarginal gyrus 3 B | 79 | Right superior parietal 3 E |
| 22 | Left supramarginal gyrus 5 A | 80 | Right superior parietal 3 F |
| 23 | Left superior parietal 2 C | 81 | Right somatosensory F |
| 24 | Left superior parietal 2 D | 82 | Right somatosensory G |
| 25 | Left superior parietal 3 A | 83 | Right somatosensory K |
| 26 | Left superior parietal 3 B | 84 | Right anterior motor B |
| 27 | Left superior parietal 4 D | 85 | Right anterior motor D |
| 28 | Left somatosensory A | 86 | Right posterior motor I |
| 29 | Left somatosensory I | 87 | Right posterior motor K |
| 30 | Left somatosensory J | 88 | Right superior frontal gyrus 5 B |
| 31 | Left somatosensory L | 89 | Right superior frontal gyrus 7 B |
| 32 | Left posterior motor J | 90 | Right middle frontal gyrus 4 C |
| 33 | Left superior frontal gyrus 2 A | 91 | Right pars opercularis A |
| 34 | Left superior frontal gyrus 2 D | 92 | Right pars opercularis B |
| 35 | Left superior frontal gyrus 3 A | 93 | Right pars opercularis C |
| 36 | Left superior frontal gyrus 3 B | 94 | Right middle pars triangularis A |
| 37 | Left superior frontal gyrus 3 D | 95 | Right posterior pars triangularis A |
| 38 | Left superior frontal gyrus 4 A | 96 | Right pars orbitalis C |
| 39 | Left superior frontal gyrus 6 B | 97 | Right frontal pole 1 D |
| 40 | Left middle frontal gyrus 1 A | 98 | Right frontal pole 2 C |
| 41 | Left middle frontal gyrus 2 A | 99 | Right frontal pole 4 B |
| 42 | Left middle frontal gyrus 2 B | 100 | Right frontal pole 4 D |
| 43 | Left middle frontal gyrus 3 A | 101 | Right lateral occipitotemporal gyrus C |
| 44 | Left middle frontal gyrus 4 A | 102 | Right cingulate gyrus E |
| 45 | Left middle frontal gyrus 5 B | 103 | Right cingulate gyrus G |
| 46 | Left pars opercularis A | 104 | Right cingulate gyrus J |
| 47 | Left pars opercularis B | 105 | Right cingulate gyrus N superior |
| 48 | Left pars opercularis C | 106 | Right cingulate gyrus P |
| 49 | Left frontal pole 3 B | 107 | Right cingulate gyrus R |
| 50 | Left frontal pole 4 A | 108 | Right hippocampus body |
| 51 | Left frontal pole 4 B | 109 | Right posterior short insular gyrus C |
| 52 | Left lateral occipitotemporal gyrus C | 110 | Left caudate |
| 53 | Left cingulate gyrus G | 111 | Left putamen |
| 54 | Left cingulate gyrus H | 112 | Left pallidum |
| 55 | Left cingulate gyrus J | 113 | Right thalamus |
| 56 | Left cingulate gyrus Q | 114 | Left cerebellum 3 |
| 57 | Left anterior short insular gyrus A | 115 | Left cerebellum 10 |
| 58 | Left posterior short insular gyrus A |  |  |

Note that anatomical locations of each region are available at https://yalebrainatlas.github.io/YaleBrainAtlas/.

**Supplementary table 4**. Univariate and multivariate Cox proportional hazards model estimating the hazard ratio (HR) of cognitive impairment in class I: general cognitive ability, class II: verbal ability, and class III: non-verbal ability.

| **Hazards**  **model** | **Model**  **type** | **Indicator** | | **Class I** | | | | **Class II** | | | | **Class III** | | | |
| --- | --- | --- | --- | --- | --- | --- | --- | --- | --- | --- | --- | --- | --- | --- | --- |
|  |  |  |  | **HR**  **(95% CI)** | **p-value** | **Model Performance** | | **HR**  **(95% CI)** | **p-value** | **Model Performance** | | **HR**  **(95% CI)** | **p-value** | **Model Performance** | |
|  |  |  |  |  |  | **AIC** | **bias-corrected**  **C-index** |  |  | **AIC** | **bias -corrected**  **C-index** |  |  | **AIC** | **bias -corrected**  **C-index** |
| Univariate | Model 1 | Assigned GASE | | 1.15  (0.97, 1.35) | 0.103 | 79.33 | 0.618 | 1.16  (1.02, 1.31) | 0.022* | 89.83 | 0.641 | 1.25  (0.96, 1.63) | 0.100 | 37.98 | 0.653 |
|  | Model 2 | Predicted GASE | | 1.19  (1.02, 1.39) | 0.028* | 76.69 | 0.719 | 1.18  (1.05, 1.33) | 0.006** | 87.19 | 0.717 | 1.26  (0.99, 1.61) | 0.065 | 37.20 | 0.637 |
| Multivariate | Model 1 | Assigned GASE | | 1.12  (0.93, 1.35) | 0.215 | 86.20 | 0.518 | 1.17  (1.02, 1.34) | 0.025* | 96.85 | 0.582 | 1.34  (0.99, 1.81) | 0.057 | 44.14 | 0.614 |
|  |  | Non-lesional | Invisible | Used as reference |  |  |  |  |  |  |  |  |  |  |  |
|  |  | FCD | Extra-temporal | 1.71  (0.39, 7.53) | 0.478 |  |  | 0.70  (0.19, 2.60) | 0.597 |  |  | 0.50  (0.04, 6.46) | 0.596 |  |  |
|  |  |  | Temporal | 1.13  (0.25, 5.13) | 0.873 |  |  | 0.80  (0.19, 3.32) | 0.755 |  |  | 2.24  (0.35, 14.21) | 0.391 |  |  |
|  |  | Other lesions  (tumor, HS, Ence) | Extra-temporal | 0.79  (0.08, 7.77) | 0.840 |  |  | 0.77  (0.09, 6.94) | 0.815 |  |  | 1.78  (0.14, 23.21) | 0.660 |  |  |
|  |  |  | Temporal | 1.81  (0.44, 7.48) | 0.415 |  |  | 1.38  (0.37, 5.17) | 0.629 |  |  | 1.47  (0.19, 11.22) | 0.709 |  |  |
|  | Model 2 | Predicted GASE | | 1.23  (1.03, 1.48) | 0.022* | 81.65 | 0.686 | 1.22  (1.07, 1.40) | 0.003** | 92.26 | 0.685 | 1.33  (1.02, 1.74) | 0.036* | 43.15 | 0.626 |
|  |  | Non-lesional | Invisible | Used as reference |  |  |  |  |  |  |  |  |  |  |  |
|  |  | FCD | Extra-temporal | 1.57  (0.36, 6.80) | 0.546 |  |  | 0.64  (0.17, 2.44) | 0.515 |  |  | 0.58  (0.05, 7.49) | 0.679 |  |  |
|  |  |  | Temporal | 0.98  (0.21, 4.55) | 0.982 |  |  | 0.66  (0.16, 2.80) | 0.573 |  |  | 1.78  (0.27, 11.80) | 0.549 |  |  |
|  |  | Other lesions  (tumor, HS, Ence) | Extra-temporal | 0.48  (0.05, 4.51) | 0.517 |  |  | 0.41  (0.05, 3.65) | 0.425 |  |  | 0.60  (0.05, 7.14) | 0.690 |  |  |
|  |  |  | Temporal | 2.58  (0.58, 11.54) | 0.216 |  |  | 1.88  (0.48, 7.33) | 0.363 |  |  | 2.34  (0.28, 19.44) | 0.432 |  |  |

FCD: focal cortical dysplasia; HS: hippocampal sclerosis; Ence: encephalitis; *: p-value < 0.05; **: p-value < 0.01; ***: p-value < 0.001.
